# How COVID-19 Has Influenced Public Interest in Antimicrobials, Antimicrobial Resistance and Related Preventive Measures: A Google Trends Analysis of Italian Data

**DOI:** 10.3390/antibiotics11030379

**Published:** 2022-03-13

**Authors:** Andrea Maugeri, Martina Barchitta, Guido Basile, Antonella Agodi

**Affiliations:** 1 Department of Medical and Surgical Sciences and Advanced Technologies “GF Ingrassia”, University of Catania, Via S. Sofia 87, 95123 Catania, Italy; andrea.maugeri@unict.it (A.M.); martina.barchitta@unict.it (M.B.); 2 Department of General Surgery and Medical-Surgical Specialties, University of Catania, Via S. Sofia 78, 95123 Catania, Italy; gbasile@unict.it

**Keywords:** antibiotic resistance, Google analytics, handwashing, preventive measures, hand hygiene, disinfectant

## Abstract

Google Trends analytics is an innovative way to evaluate public interest in antimicrobial resistance (AMR) and related preventive measures. In the present study, we analyzed Google Trends data in Italy, from 2016 to 2021. A joinpoint analysis was performed to assess whether and how annual campaigns and the COVID-19 pandemic affected public interest in antimicrobials, AMR, hand hygiene, and the use of disinfectant. For the terms “antimicrobials” and “antimicrobial resistance”, no joinpoints were detected around the time of the World Antimicrobial Awareness Week. Similarly, the COVID-19 pandemic seems to have had no effect on public interest in this term. For the term “handwashing”, no joinpoints were detected around World Hand Hygiene Day or Global Handwashing Day. However, three joinpoints were detected around the peak of interest observed in March 2020, after the beginning of the COVID-19 pandemic. Comparable results were obtained for the term “disinfectant”. These findings show that the influence of annual campaigns on public interest in AMR and preventive measures was modest and not long-term. The COVID-19 pandemic, meanwhile, had no effect on AMR but raised awareness on preventive measures. However, this was a temporary rather than long-term outcome. Thus, different policies, strategies, and measures should be designed to advocate prevention of AMR in the COVID-19 era.

## 1. Introduction

Antimicrobial resistance (AMR) is a threat to human health that is becoming more serious every year, as demonstrated by the 670,000 infections and 33,000 deaths caused by AMR bacteria in Europe in 2020 [1]. At present, AMR accounts for a burden similar to that of influenza, tuberculosis, and HIV-AIDS combined [1]. The situation may become more complicated because AMR is estimated cause more deaths than cancer by 2050 [2]. As such, the World Health Organization (WHO) established the World Antimicrobial Awareness Week (WAAW), held annually since 2015, to raise awareness of AMR and encourage best practices to counteract its spread [3]. Projections for AMR do not account for the possible impact of COVID-19 on AMR. The current pandemic has placed huge strains on health systems and resources globally. A concerning long-term consequence of the COVID-19 pandemic is the potential spread of AMR due to an increased and often inappropriate use of antimicrobials [4]. 

The pandemic has emphasized the importance of measures for the prevention and control of infections at the public level [4]. Among these measures, hand hygiene is considered one of the most effective actions for preventing and controlling infections. Accordingly, the WHO established World Hand Hygiene Day (WHHD), held annually since 2009, to promote hand hygiene practices in all healthcare facilities [5]. With a similar scope, the Global Handwashing Partnership, which brings together private sector entities, academic institutions, and governmental and community-based organizations, established Global Handwashing Day (GHD) in 2008 [6]. Beyond hand hygiene, other preventive measures have become a part of current life as a result of the COVID-19 pandemic. These measures include social distancing and the use of surgical masks and disinfectant [4]. 

Recently, increasing public interest in preventive measures might help counteract both AMR and the COVID-19 pandemic. However, although some of these preventive measures have proven to be effective against AMR and the COVID-19 pandemic, public interest and awareness on these themes differ from country to country [7,8,9,10]. In this context, infodemiology, the science of monitoring, interpreting, and managing web-based information on health and disease [11], is proving to be a useful instrument for public health professionals. Digital surveillance, also called infoveillance, is attracting widespread interest because of its potential to monitor diseases and evaluate Internet search behaviors related to health issues [11,12,13,14]. As a part of infodemiology, Google Trends analytics can be applied in the field of infectious diseases to anticipate outbreaks, model epidemics, and evaluate public awareness on preventive measures [15,16,17,18,19,20,21,22,23].

In our study, we monitored public interest in antimicrobials, AMR, and related preventive measures using Google Trends data. In particular, we evaluated whether and, if so, how annual campaigns (i.e., WAAW and WHHD) and the COVID-19 pandemic affected public interest regarding antimicrobials, AMR, hand hygiene, and the use of disinfectant in Italy. Among European countries, Italy is characterized by a high number of COVID-19 infections and AMR at both the national and regional level [24,25,26,27,28], and it is one of the countries most severely affected by the COVID-19 pandemic [29,30]. 

## 2. Results

### 2.1. Public Interest in Antimicrobials and Antimicrobial Resistance

**Figure 1:**
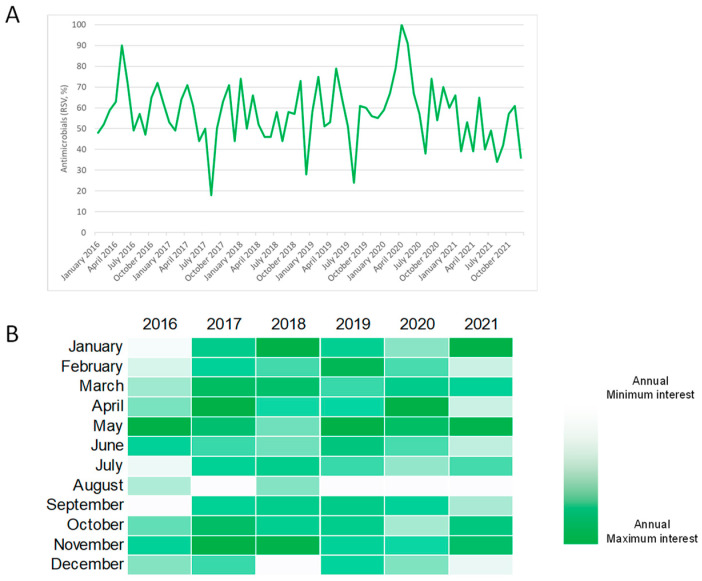
Public interest in the term “antimicrobials” based on Google Trends data. (**A**) Relative search volume over time from January 2016 to December 2021. (**B**) Heatmap showing the intensity of monthly public interest per year.

Figure 1A illustrates the monthly public interest in the term “antimicrobials” from 1 January 2016 to 31 December 2021. For each year, the annual peak of interest occurred in May 2016 and 2019, November 2017 and 2018, April 2020, and January 2021. Overall, public interest reached its peak in April 2020. However, no significant joinpoints were detected in this time series and the analysis of weekly trends in public interest produced similar results (data not shown).

**Figure 2:**
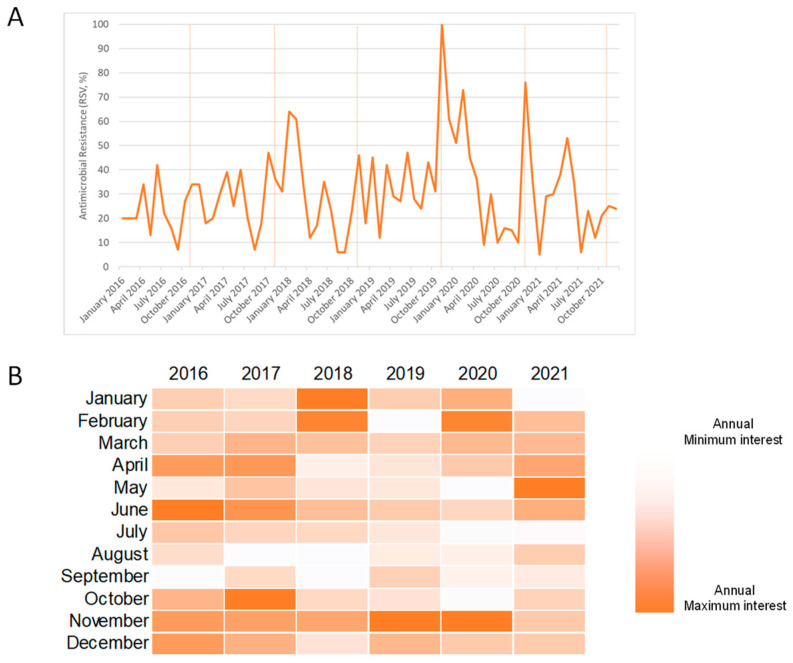
Public interest in the term “antimicrobial resistance” based on Google Trends data. (**A**) Relative search volume over time from January 2016 to December 2021. Vertical orange lines correspond to the months when WAAWs occurred; (**B**) heatmap showing the intensity of monthly public interest per year.

**Figure 3:**
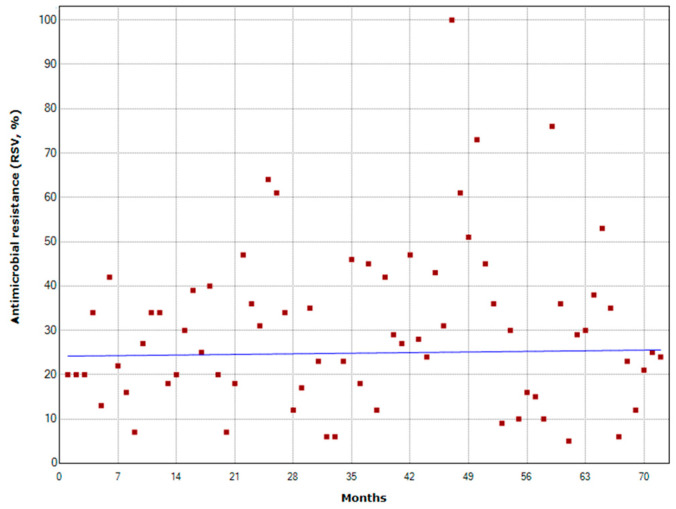
Joinpoint analysis of public interest in the term “antimicrobial resistance”.

Figure 2A illustrates monthly public interest in the term “antimicrobial resistance” from 1 January 2016 to 31 December 2021. For each year, the annual peak of interest occurred in June 2016, October 2017, January 2018, November 2019 and 2020, and May 2021 (Figure 2B). The WAAW is celebrated from 18 to 24 November each year. Overall, public interest reached its peak in November 2019. However, no significant joinpoints were detected in this time series (Figure 3) and the analysis of weekly trends in public interest produced similar results (data not shown).

### 2.2. Public Interest in Preventive Measures

**Figure 4:**
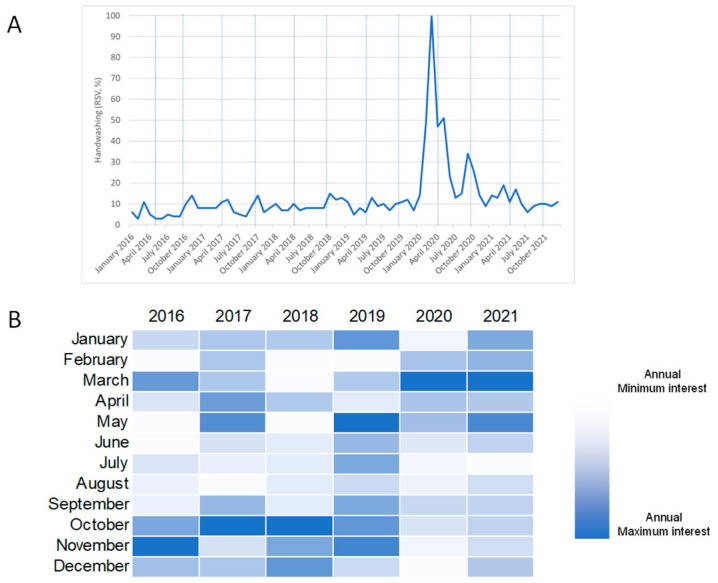
Public interest in the term “handwashing” based on Google Trends data. (**A**) Relative search volume over time from January 2016 to December 2021. Vertical blue lines correspond to the months when annual campaigns occurred; (**B**) heatmap showing the intensity of monthly public interest per year.

**Figure 5:**
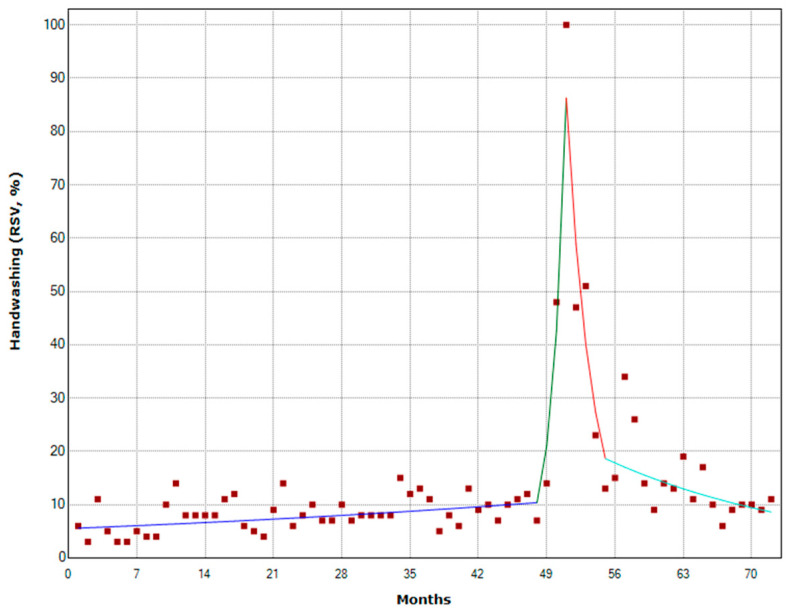
Joinpoint analysis of public interest in the term “handwashing”.

Figure 4A illustrates monthly public interest in the term “handwashing” from 1 January 2016 to 31 December 2021. For each year, the annual peak of interest occurred in November 2016, October 2017 and 2018, May 2019, and February 2020 and 2021 (Figure 4B). The WHHD is celebrated on 5 May, while GHD is on 15 October. Overall, public interest reached its peak in March 2020 and the analysis detected three joinpoints around this peak (Figure 5). From January 2016 to December 2019, public interest remained relatively stable (MPC = 1.3%) and then sharply increased up to a peak in March 2020 (MPC = 102.6%). Then, public interest rapidly declined until August 2020 (MPC = −31.8%), and then slowed until December 2021 (MPC = −4.4%). The analysis of weekly trends in public interest produced similar results (data not shown).

**Figure 6:**
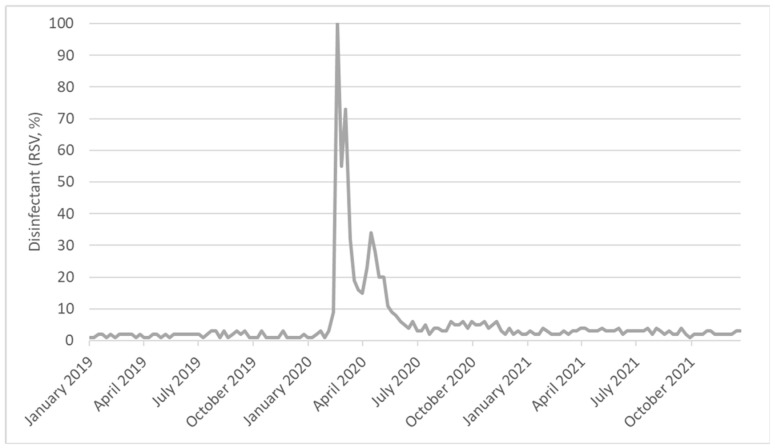
Public interest in the term “disinfectant” based on Google Trends data.

Figure 6 illustrates weekly public interest in the term “disinfectant” from 1 January 2019 to 31 December 2021. The analysis was limited to this period since public interest was extremely low before 2019. In general, interest in this term was low until early 2020 and then increased in the following months. In particular, public interest remained stably low until the 57th week (26 January–2 February 2020; WPC = −0.3%), but then rapidly increased until its highest peak in the 62nd week (1–8 March 2020; WPC = 246.4%). Since that point, public interest decreased moderately at first (until 28 June–5 July 2020; WPC = −13.1%) and then remained stable until the end of 2021 (WPC = −0.1%).

### 2.3. Correlations between Google Trends and COVID-19 Cases

Finally, we evaluated correlations between Google Trends data and percentage changes in COVID-19 cases from March 2020 to December 2021. At the monthly level, no significant correlations of public interest in “antimicrobials”, “antimicrobial resistance”, and “handwashing” with percentage changes in COVID-19 cases were evident (*p*-values = 0.974, 0.966 and 0.439, respectively). In contrast, we found a negative but weak correlation with weekly public interest in “handwashing” (rho = −0.248; *p* = 0.015). 

## 3. Discussion

In the present study, we sought to determine the level of public interest in AMR in Italy, as well as the potential effects of WAAW and COVID-19 on Google search activity. Before the COVID-19 epidemic, public interest in AMR reached its greatest value in November 2019, the month in which the WAAW is celebrated (i.e., from 18 to 24 November) [3]. A high value was also observed in November 2020, but this effect was not permanent. Regarding interest in the term “antimicrobials”, high values were found in November 2017 and 2018, even though the highest peak was reached in April 2020. Our findings, therefore, suggest that the WAAWs did not lead to significant changes in public interest annually and that their effect was generally modest. No joinpoints were detected around the WAAW from 2016 to 2021. Similarly, no joinpoints were detected corresponding to the beginning of the COVID-19 pandemic. These findings are partially in line with those reported by a previous analysis using Google Trends in Japan, the U.K., the USA, and worldwide [7]. We found that the WAAW did not influence public interest in AMR at the national level. However, we observed a significant global effect of the WAAW in 2017 and 2020. Moreover, we observed a decline in public interest in AMR after the beginning of the COVID-19 pandemic. This evidence suggests, on the one hand, that the WAAW does not contribute much to an increase in awareness of AMR and, on the other hand, that the COVID-19 pandemic may have had a negative impact. Thus, the COVID-19 pandemic could have contributed to reducing the information available, measures implemented, research conducted, and general interest in future pandemics in terms of the multi-drug-resistant infections, which are already plaguing several Mediterranean countries [25,26,31,32,33,34]. For these reasons, there is a need for novel strategies to increase the interest of the general public and healthcare professionals, especially in countries where AMR rates are high. Lack of awareness of AMR and the inappropriate use of antimicrobials are the main causes of AMR [35,36,37,38]. A systematic review pointed out a low level of awareness in the general public, providing themes and insights to be addressed [39]. A recent survey of healthcare workers, launched by the European Centre for Disease Prevention and Control in 2019, gave a thorough description of their knowledge, attitudes, and behaviors regarding AMR and antimicrobial use [40,41,42,43,44]. In Italy, awareness of AMR among healthcare workers was generally low and extremely varied across professional groups [45]. Moreover, studies that analyzed preventive measures in healthcare workers during the pandemic have reported poorer results [46,47]. Thus, the current picture suggests that additional campaigns should be developed to counteract the negative effects of COVID-19. Some attempts have already been carried out at a regional level, using interactive websites and social networks [48]. However, further efforts are needed to raise awareness of AMR and antimicrobial use in the general public and among healthcare professionals.

As part of our study, we also assessed the level of public interest in another sensitive topic: hand hygiene. It is one of the key measures against AMR and COVID-19. Except for 2020, high levels of interest were reported in May and October, months in which WHHD and GHD are celebrated. Despite that, the peak of interest in hand hygiene was observed in March 2020, after the beginning of the COVID-19 pandemic in Italy. This shows how the COVID-19 pandemic emphasized the importance of hand hygiene in the general public. Accordingly, we detected three joinpoints from December 2019 to August 2020, which determined the peak observed in March 2020. In contrast, no joinpoints were evident around the WHHD or GHD. These results are similar to what was observed in Japan, the U.K., and the USA in a previous study [49]. We, did not detect joinpoints around the WHHD or GHD at the national level. However, joinpoints were noted worldwide at around the time of the WHHD and GHD, but only before the beginning of the COVID-19 pandemic [49]. 

The COVID-19 pandemic also raised public interest in other preventive measures against the pandemic, which were mostly unknown before 2020 (i.e., wearing a mask, social distancing, and the use of disinfectant). Some of these, such as the use of disinfectant, also prove useful in preventing AMR. In our study, public interest in the term “disinfectant” exhibited a trend similar to that observed for hand hygiene. Public interest was stable until February 2020, then rapidly increased until it reached its peak at the beginning of March 2020, and finally decreased until the end of 2021. A previous study in the Philippines predicted such an effect of the COVID-19 pandemic on public interest with regard to some preventive measures [8]. However, other authors also observed a positive correlation between Google Trends data and the number of new daily cases of COVID-19 [8]. In our study, we instead observed negative but weak correlations between public interest in hand hygiene and the percentage change in COVID-19 cases. This inconsistency could be at least partially explained by different periods of analysis. While the study in the Philippines considered only the first four months of 2020, we extended our analysis to December 2021. Consistent with our results, a study conducted in the USA reported negative correlations between public interest in preventive measures and the rates of confirmed COVID-19 cases after stay-at-home orders [9]. 

Our study had some limitations that should be considered. First, we evaluated public interest using Google Trends as a data source. This means that our results reflected the interest of people who had access to the Internet, with a consequent potential variation in age and other user characteristics. Second, Google Trends data do not ensure full transparency and reproducibility because the search volume is calculated on the basis of nonpublic mathematical assumptions. Third, there are different terms reflecting public interest in AMR and preventive measures. Although we used the most searched terms, some findings might vary according to different terms used and/or study period considered. Moreover, our analysis was conducted on Italian data; therefore, our findings might have been affected by the epidemiological trend in Italy. 

## 4. Materials and Methods

### 4.1. Data Sources

We first evaluated public interest in antimicrobials, AMR, and hand hygiene using data related to Google searches in Italy from 1 January 2016 to 31 December 2021. Data were downloaded from the Google Trends website [50] on 1 February 2022. The search was conducted using the Italian terms “antimicrobici”, “resistenza agli antibiotici”, and “lavare le mani”, which translate to the English terms “antimicrobials”, “antimicrobial resistance”, and “handwashing”, respectively. 

We also evaluated public interest in another measure to prevent infections using the Italian term “disinfettante” (translates to “disinfectant”). In this case, the search was limited to the period from 1 January 2019 to 31 December 2021 because this term was less commonly used before the COVID-19 pandemic. 

Google Trends data were provided as their relative search volume (RSV) at a monthly or weekly level. RSV is reported as a standardized measure with a scale from 0 (no search interest) to 100 (greatest search interest). 

Data on the daily number of positive cases since the beginning of the COVID-19 pandemic in Italy were obtained from the GitHub database [51]. These data were converted into monthly and weekly percentage changes and compared to Google Trends data.

### 4.2. Statistical Analysis

First, we described public interest over the whole period of analysis. We also compared the intensity of public interest at an annual level by calculating the minimum and maximum values of interest for each year (i.e., the comparisons were showed by separate heatmaps for each term). Next, joinpoint regression models were applied to evaluate the monthly percent change (MPC) in Google Trends for the terms “antimicrobials”, “antimicrobial resistance” and “handwashing” from 1 January 2016 to 31 December 2021. Similarly, joinpoint regression models were applied to evaluate the weekly percentage change (WPC) in Google trends for the term “disinfectant”. These models enabled us to identify time points (i.e., joinpoints) during which temporal trends significantly changed. The grid search method was applied to determine the optimal number of joinpoints, from a minimum of 0 to a maximum of 3. The model selection was based on a permutation test (5000 permutations), with a significance level of 0.05. These analyses were implemented on the Joinpoint Regression Program (version 4.9.0.0; Statistical Research and Applications Branch, National Cancer Institute). Correlations between Google Trends and the monthly or weekly percentage changes in COVID-19 cases were evaluated by the Spearman’s rank order correlation test on SPSS software (version 26.0, IBM Corp., Armonk, NY, USA).

## 5. Conclusions

In conclusion, our study findings suggest that the influence of annual campaigns on public interest in antimicrobials, AMR, and hand hygiene was modest. In particular, these campaigns did not represent a long-lasting solution to raise public awareness concerning these sensitive topics. On the contrary, the effect of the COVID-19 pandemic was significant. It raised awareness on preventive measures against AMR, such as hand hygiene and the use of disinfectant. After an initial increase due to the pandemic, however, public interest on preventive measures decreased again. For these reasons, additional and different policies, strategies, and measures should be designed to advocate prevention of AMR in the COVID-19 era.

## Data Availability

Not applicable.
